# Targeting the Golgi apparatus to overcome acquired resistance of non-small cell lung cancer cells to EGFR tyrosine kinase inhibitors

**DOI:** 10.18632/oncotarget.22895

**Published:** 2017-12-06

**Authors:** Yoshimi Ohashi, Mutsumi Okamura, Ryohei Katayama, Siyang Fang, Saki Tsutsui, Akinobu Akatsuka, Mingde Shan, Hyeong-Wook Choi, Naoya Fujita, Kentaro Yoshimatsu, Isamu Shiina, Takao Yamori, Shingo Dan

**Affiliations:** ^1^ Division of Molecular Pharmacology, Cancer Chemotherapy Center, Japanese Foundation for Cancer Research, Tokyo, Japan; ^2^ Division of Experimental Chemotherapy, Cancer Chemotherapy Center, Japanese Foundation for Cancer Research, Tokyo, Japan; ^3^ Eisai AiM Institute, Eisai Inc., Andover, MA, USA; ^4^ Tsukuba Research Labs., Eisai Co., Ltd., Tokyo, Japan; ^5^ Department of Applied Chemistry, Faculty of Science, Tokyo University of Science, Tokyo, Japan; ^6^ Present address: Center for Product Evaluation, Pharmaceuticals and Medical Devices Agency, Tokyo, Japan

**Keywords:** Golgi apparatus, ADP ribosylation factor 1 (Arf1), non-small cell lung cancer (NSCLC), epidermal growth factor receptor (EGFR), acquired resistance

## Abstract

Epidermal growth factor receptor (EGFR)-tyrosine kinase inhibitors (EGFR-TKIs) were demonstrated to provide survival benefit in patients with non-small cell lung cancer (NSCLC) harboring activating mutations of EGFR; however, emergence of acquired resistance to EGFR-TKIs has been shown to cause poor outcome. To overcome the TKI resistance, drugs with different mode of action are required. We previously reported that M-COPA (2-methylcoprophilinamide), a Golgi disruptor, suppressed the growth of gastric cancers overexpressing receptor tyrosine kinases (RTKs) such as hepatocyte growth factor receptor (MET) *via* downregulating their cell surface expression. In this study, we examined the antitumor effect of M-COPA on NSCLC cells with TKI resistance. As a result, M-COPA effectively downregulated cell surface EGFR and its downstream signals, and finally exerted *in vivo* antitumor effect in NSCLC cells harboring secondary (T790M/del19) and tertiary (C797S/T790M/del19) mutated EGFR, which exhibit acquired resistance to first- and third generation EGFR-TKIs, respectively. M-COPA also downregulated MET expression potentially involved in the acquired resistance to EGFR-TKIs *via* bypassing the EGFR pathway blockade. These results provide the first evidence that targeting the Golgi apparatus might be a promising therapeutic strategy to overcome the vicious cycle of TKI resistance in EGFR-mutated NSCLC cells *via* downregulating cell surface RTK expression.

## INTRODUCTION

Lung cancer is the first leading cause of cancer death worldwide, and the five-year survival rate is 4–17% depending on the clinical stage and research area [1]. One reason for this poor prognosis is that non-small cell lung cancer (NSCLC) has become resistant to platinum-based traditional chemotherapy [2]. Activating mutations of epidermal growth factor receptor (EGFR) such as the L858R point mutation and small in-frame deletions in the region encoded by exon 19 (del19) occur in 11–17% of patients with NSCLC [1, 3], and notably in about 40% of Asian patients [4]. In these patients, the growth and survival of tumor cells have become exclusively dependent on the aberrant activation of EGFR, termed an ‘*oncogene-addicted*’ state [5], and therefore, activated EGFR is an ideal therapeutic target. First-generation EGFR tyrosine kinase inhibitors (EGFR-TKIs) including gefitinib and erlotinib were developed and have antitumor activity and survival benefit in these patients [6–8]. However, unavoidable acquired drug resistance to EGFR-TKI has been reported [9–11]. The most common mechanism of acquired resistance to TKIs is caused by a secondary mutation on the gatekeeper residue of the ATP-binding pocket of EGFR (T790M). To overcome this resistance, more potent irreversible TKIs including afatinib and drugs that selectively target the EGFR-T790M mutant such as osimertinib and rociletinib have been developed as second- and third-generation EGFR-TKIs, respectively. However, the emergence of a secondary (T790M) and tertiary (C797S) dual mutant that exhibits resistant to all these TKIs was recently reported [12, 13]. Another mechanism involves the activation of an alternative signaling pathway that bypasses EGFR; for example, the gene amplification of MET (hepatocyte growth factor (HGF) receptor) or its ligand HGF [14]. To overcome this form of resistance, MET-TKIs such as crizotinib and INC280 in combination with an EGFR-TKI have been tested in clinical trials (https://clinicaltrials.gov:NCT01610336).

The Golgi apparatus is an intracellular organelle with a central role in the transport, processing, and sorting of secreted and plasma membrane proteins [15–17]. We previously demonstrated that M-COPA (2-methylcoprophilinamide, previously termed ‘*AMF-26*’), disrupted the Golgi apparatus structure by inhibiting the activation of ADP ribosylation factor 1 (Arf1), and finally exhibited antitumor effect *in vitro* and *in vivo*, like another well-characterized Arf1 inhibitor brefeldin A (BFA) did [18–22]. Arf1 is an isoform of class I Arfs that regulate the formation of COPI and/or clathrin-coated transport vesicles from the endoplasmic reticulum (ER) through the Golgi to the cell surface [23–27]. In tumor cells, gene amplification as well as activating somatic mutations of RTKs represented by an activating mutation of EGFR in NSCLC cells, is often observed and the growth and survival of these tumor cells sometimes become exclusively dependent on (or ‘*addicted to*’) the aberrant overexpression of the RTK protein expressed on the cell surface [11, 28–32]. Because the Golgi apparatus regulates the transport of RTKs onto the cell surface, we have focused on the effect of M-COPA on RTK-addicted tumor cells. We previously demonstrated that M-COPA effectively downregulated the cell surface expression of MET and showed preferential antitumor efficacy in MET-amplified gastric cancer cells both *in vitro* and *in vivo* [33]. These results prompted us to examine whether M-COPA might also be effective against tumor cells harboring an activating somatic mutation in a specific RTK gene.

In the present study, we examined the effect of M-COPA on NSCLC cells harboring an EGFR activating mutation, especially those exhibiting acquired resistance to EGFR-TKIs. We report the first evidence that M-COPA has a preferential antitumor effect on NSCLC cells harboring activating L858R and del19 mutations, but also those with a T790M/del19 double mutation and C797S/T790M/del19 triple mutation, which exhibit resistance to first- and third-generation EGFR-TKIs, respectively. M-COPA markedly downregulated the cell surface expression of EGFR irrespective of its mutation status, and also downregulated MET expression exclusively observed in EGFR-TKI-resistant cells. These results suggest that Golgi-targeted drugs might provide a novel therapeutic option for treating EGFR-activated NSCLC cells, and especially for overcoming TKI resistance by multiple mechanisms, by downregulating the cell surface expression of both mutated EGFR and MET involved in the EGFR-bypassing alternative pathway.

## RESULTS

### M-COPA inhibits the cell surface expression of EGFR and EGFR-downstream signal transduction pathways in NSCLC cell lines harboring an activating EGFR mutation

**Figure 1:**
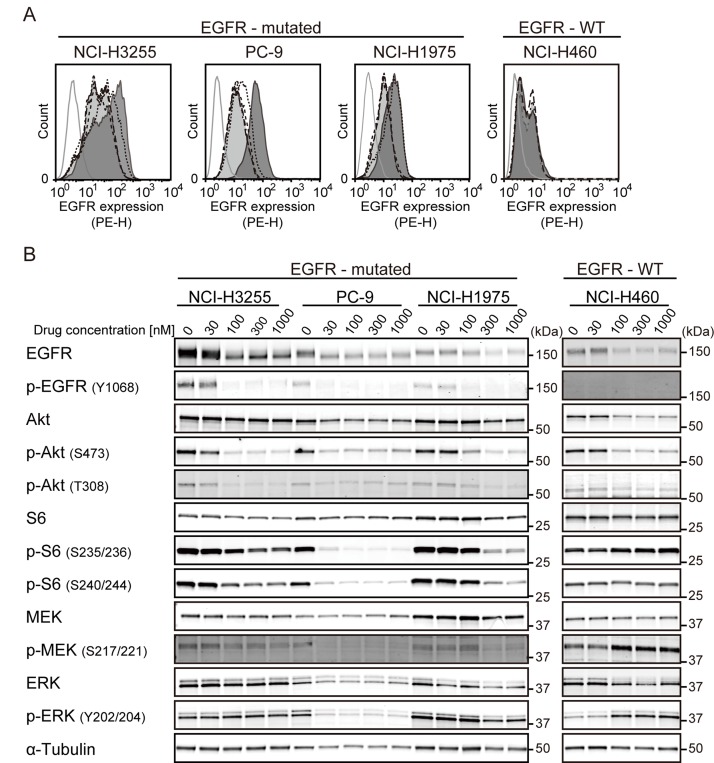
M-COPA downregulates cell surface EGFR and its downstream signaling in EGFR-addicted cell lines (**A**) EGFR expression on the cell surface was measured by FCM analysis. Cells were treated with M-COPA at the indicated concentrations for 24 h, and stained with a PE-conjugated anti-EGFR antibody. Lines and areas are used to indicate drug concentrations: black solid lines with dark gray area, no drug; black dotted lines, 30 nM; black dashed lines, 100 nM; black long dashed lines, 300 nM; black chain lines with light gray area, 1000 nM; and gray solid lines, stained with isotype-control IgG. Experiments were performed at least twice and representative results are indicated. (**B**) Expression levels of total proteins and the phosphorylated forms of EGFR signaling molecules, including Akt, ribosomal S6 protein (S6), MEK, and ERK were examined by immunoblot analysis, upon treatment with M-COPA. Cells were treated with M-COPA at the indicated concentrations for 24 h, and cell extracts were prepared. Proteins in the cell extract were separated by SDS-PAGE and electroblotted onto a membrane. The membrane was then probed with antibodies against the indicated proteins. Experiments were performed at least twice and representative results are indicated.

First, we examined the effect of M-COPA treatment on the cell surface expression of EGFR protein in NSCLC cell lines harboring an activating EGFR mutation by FCM analysis. As shown in Figure 1A, the cell surface expression of EGFR was detected in the NSCLC cell lines NCI-H3255 (L858R), PC-9 (del19) and NCI-H1975 (T790M/L858R), and the expression levels were decreased in a dose-dependent manner upon treatment with M-COPA. In contrast, the baseline expression of EGFR at the cell surface was relatively low in NCI-H460 (EGFR-wild type and KRAS mutated) and its expression was only slightly affected upon treatment with M-COPA

To clarify the effect of M-COPA on EGFR-downstream signal transduction pathways, we examined the expression levels of total proteins and phosphorylated forms of EGFR itself, Akt-mTOR pathway factors (Akt and ribosomal S6 protein) and MEK-ERK pathway factors (MEK and ERK) by immunoblot analyses. As shown in Figure 1B, the phosphorylated forms of EGFR, Akt (S473 and T308), S6 (S235/236 and S240/244), MEK and ERK were markedly reduced at an M-COPA concentration of 30 nM or higher in PC-9 cells, and 100 nM or higher in NCI-H3255 and NCI-H1975 cells, respectively. This finding paralleled the downregulation of cell surface EGFR (Figure 1A). In contrast, the phosphorylated form of S6 remained unchanged and phosphorylated MEK and ERK were upregulated in EGFR-wild type NCI-H460 cells. These data suggest that the downregulation of cell surface EGFR upon M-COPA treatment attenuates the Akt-mTOR and MEK-ERK pathways selectively in NSCLC cells harboring an activating EGFR mutation.

### M-COPA inhibits the growth of NSCLC cell lines

**Figure 2:**
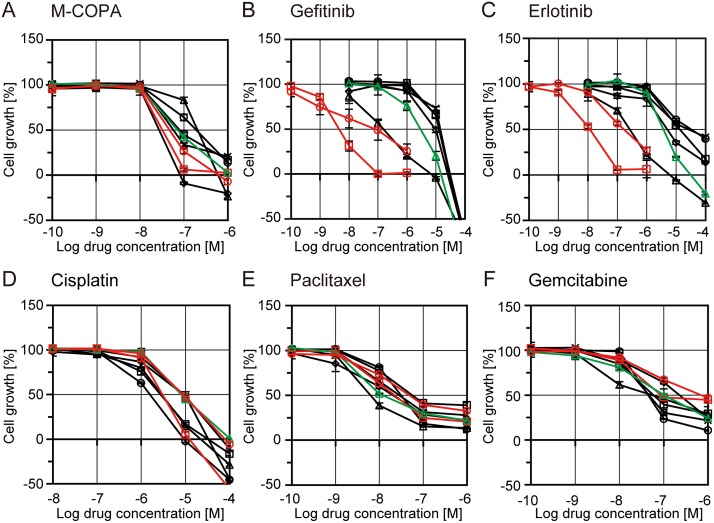
Growth inhibitory activities of M-COPA, EGFR-TKIs, and chemotherapeutic agents in human NSCLC cell lines with or without EGFR-activating mutations NSCLC cell lines were treated with drugs in a series of 5-dose 10-fold dilutions at the indicated concentration range for 48 h. Cells were then fixed and stained by sulforhodamine B (SRB) assay. Growth inhibition curves of M-COPA (**A**), gefitinib (**B**), erlotinib (**C**), cisplatin (**D**), paclitaxel (**E**) and gemcitabine (**F**) are shown. Symbols indicate the following: EGFR-WT cell lines, black square, NCI-H23; black triangle, NCI-H522; black rhombus, NCI-H226; black circle, NCI-H460; black x-mark, A549. EGFR-activated cell lines, red circle, NCI-H3255 (L858R); red square, PC-9 (del19); and green triangle, NCI-H1975 (T790M/L858R). Experiments were performed at least twice and representative results are indicated.

Next, we evaluated the effect of M-COPA on the growth of NSCLC cell lines with or without an EGFR activating mutation, and compared it with the effect of EGFR-TKIs. In agreement with previous reports [34, 35], two EGFR-activated cell lines, NCI-H3255 (L858R) and PC-9 (del19), were highly sensitive to the first-generation EGFR inhibitors, gefitinib and erlotinib (Figure 2B and 2C), when compared with five other NSCLC cell lines carrying wild type EGFR. In contrast, NCI-H1975, a cell line derived from an EGFR-TKI-naïve NSCLC patient that intrinsically harbors an EGFR-T790M gatekeeper mutation in the ATP-binding pocket in addition to an activating mutation (L858R) [36], was resistant to gefitinib and erlotinib (Figure 2B and 2C), as reported previously [37]. Regarding M-COPA, the EGFR-activated cell lines, including NCI-H1975, exhibited a better drug response than those with the EGFR WT gene (Figure 2A). The only exception was that NCI-H226 tended to be highly sensitive. However, the selectivity was not as marked as that observed for the first-generation EGFR-TKIs. For comparison, the anticancer effects of chemotherapeutic agents currently used in NSCLC therapy, namely cisplatin, paclitaxel and gemcitabine, were examined. None of these drugs exhibited significant differences in terms of inhibition of the growth of NSCLC cell lines, irrespective of their EGFR mutation status (Figure 2D–2F).

### Antitumor effect of M-COPA on the first-generation EGFR-TKI-resistant NSCLC cells harboring EGFR with a secondary T790M mutation

**Figure 3:**
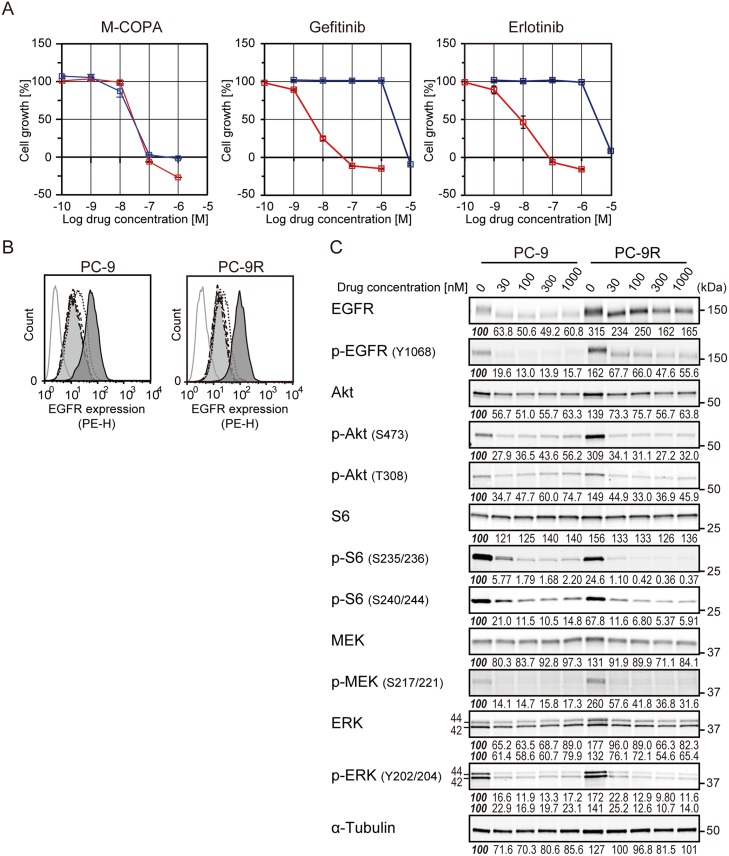
The effect of M-COPA and first-generation EGFR-TKIs on cell growth, cell surface expression of EGFR and its downstream signaling in PC-9 and its gefitinib resistant subline PC-9R (**A**) Growth inhibitory activities of M-COPA, gefitinib and erlotinib in the EGFR-activated NSCLC cell line, PC-9 (del19), and gefitinib-resistant PC-9R (T790M/del19). Growth inhibition assays after treatment with M-COPA, gefitinib or erlotinib were performed as described in Figure 2. Symbols indicate the following: red square, PC-9; blue square, PC-9R. (**B**) EGFR expression on the cell surface was measured by FCM analysis. Lines and areas are described in the Figure 1A legend. (**C**) Expression levels of total proteins and the phosphorylated forms of EGFR signaling molecules in PC-9 and PC-9R cells upon treatment with M-COPA at the indicated concentrations, as described in Figure 1B. Experiments were performed at least twice and representative results are indicated. Numbers indicated in each panel were relative intensities (%) of immunoreactive bands normalized to control sample (refereed as ‘*100%*’).

Because M-COPA was effective to EGFR-TKI-naïve NCI-H1975 cells harboring an EGFR-T790M mutation, we next investigated whether M-COPA was also effective against NSCLC cells that had acquired resistance to first-generation EGFR-TKI. We established a gefitinib-resistant PC-9 variant cell line, PC-9R (for details, see Materials and Methods). NGS analysis of the gDNA from PC-9R revealed this variant had T790M gatekeeper mutation (Supplementary Figure 1). PC-9R exhibited an acquired resistance to EGFR-TKIs, with a 50% growth inhibitory concentration value (GI_50_) of 2.88 μM and 3.47 μM to gefitinib and erlotinib, respectively, whereas the growth of parental PC-9 cells were clearly suppressed at nanomolar levels (GI_50_ = 4.07 nM and 8.32 nM, respectively; hereinafter the same) (Figure 3A). In contrast, M-COPA exhibited similar inhibition of the growth of both PC-9 and PC-9R cells (34.7 nM and 33.1 nM, respectively). FCM analyses revealed that M-COPA downregulated the cell surface EGFR expression at a concentration of 30 nM or higher within 24 h of treatment (Figure 3B). Correspondingly, the active phosphorylated forms of EGFR, Akt, S6, MEK, and ERK were dramatically reduced in PC-9R as well as PC-9, despite that baseline activity of EGFR and its downstream signals were upregulated (Figure 3C). Immunofluorescent staining demonstrated that M-COPA dispersed Golgi structure in both PC-9 and PC-9R cells (50% effective concentration value, EC_50_ = 79.2 nM and 72.3 nM, respectively) within 1 h of treatment (Supplementary Figure 3). These results clearly indicate that M-COPA dispersed Golgi structure, downregulated EGFR and its downstream signals, and finally exerted a comparable antitumor effect in gefitinib-resistant PC-9R cells as in their parental PC-9 cells *in vitro*.

### Antitumor effect of M-COPA on third-generation EGFR-TKI-resistant NSCLC cells harboring EGFR with a tertiary C797S mutation

**Figure 4:**
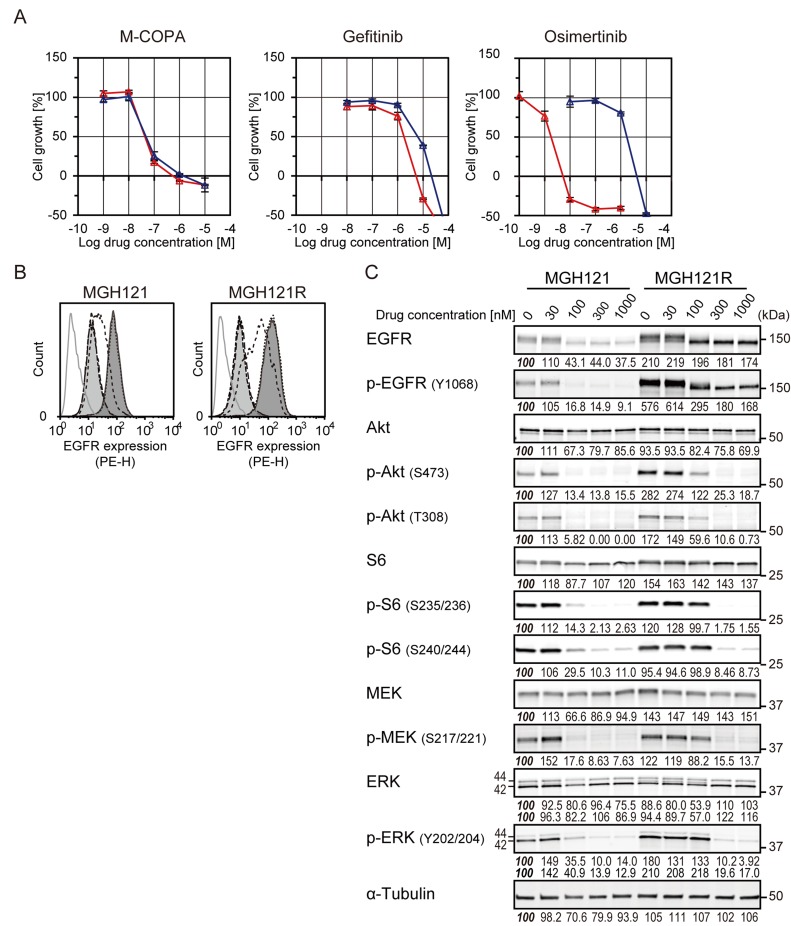
The effect of M-COPA, gefitinib and a third-generation EGFR-TKI, osimertinib, on cell growth, cell surface expression of EGFR and downstream signaling in MGH121 and its osimertinib-resistant subline MGH121R (**A**) Growth inhibitory activities of M-COPA, gefitinib and osimertinib in a first-generation EGFR-TKI-resistant NSCLC cell line, MGH121 (T790M/del19), and third-generation EGFR-TKI-resistant MGH121R (C797S/T790M/del19). Growth inhibition assays upon treatment with M-COPA, gefitinib or osimertinib were performed as described in Figure 2. Symbols indicate the following: red triangle, MGH121; blue triangle, MGH121R. (**B**) EGFR expression on the cell surface was measured by FCM analysis. Lines and areas are described in Figure 1A legend. (**C**) Expression levels of total proteins and the phosphorylated forms of EGFR signaling molecules in MGH121 and MGH121R cells upon treatment with M-COPA at the indicated concentrations are described in Figure 1B. Experiments were performed at least twice and representative results are indicated. Numbers indicated in each panel were relative intensities (%) of immunoreactive bands normalized to control sample (refereed as ‘*100%*’).

Because M-COPA had a potent antitumor effect on NSCLC cells with acquired resistance to first-generation EGFR-TKIs, we next investigated the antitumor activity of M-COPA on NSCLC cells that had acquired resistance to a third-generation EGFR-TKIs. We utilized MGH121 and its WZ4002-resistant variant cell lines, MGH121R cell lines [13, 38]. MGH121 was originally derived from a biopsy of a resistant EGFR-mutant tumor with an EGFR-activating mutation (del19) and which had developed a T790M secondary mutation in the clinical tumor after 7 months’ administration of erlotinib. MGH121 cells were resistant to the first-generation EGFR-TKI gefitinib with a GI_50_ concentration of 1.78 μM, consistent with the presence of a T790M resistance mutation; however, it was highly sensitive to the third-generation TKI osimertinib with a GI_50_ concentration of 1.78 nM (Figure 4A). In contrast, MGH121R variant cells, harboring a tertiary C797S mutation in addition to T790M, were resistant to both gefitinib and osimertinib (6.17 μM and 1.74 μM, respectively) (Figure 4A). We then examined the antitumor effect of M-COPA against these cell lines. As expected, M-COPA exhibited an inhibitory activity on the growth of MGH121R cells (46.8 nM) comparable to the parental MGH121 cells (43.7 nM) and the other EGFR-activating NSCLC cells examined above. Correspondingly, M-COPA downregulated the cell surface EGFR expression and phosphorylated forms of EGFR, Akt, S6, MEK, and ERK, upregulated in MGH121R cells (Figure 4B and 4C). Immunofluorescent staining demonstrated that M-COPA dispersed Golgi structure of MGH121 and MGH121R cells (EC_50_ = 248 nM and 397 nM, respectively; Supplementary Figure 3). From these results, we concluded that M-COPA exerts an antitumor effect on EGFR-activating NSCLC cells, irrespective of the presence of secondary or tertiary acquired mutations.

### Inhibitory effect of M-COPA on EGFR-bypassing MET overexpression in EGFR-TKI-resistant NSCLC cell lines

**Figure 5:**
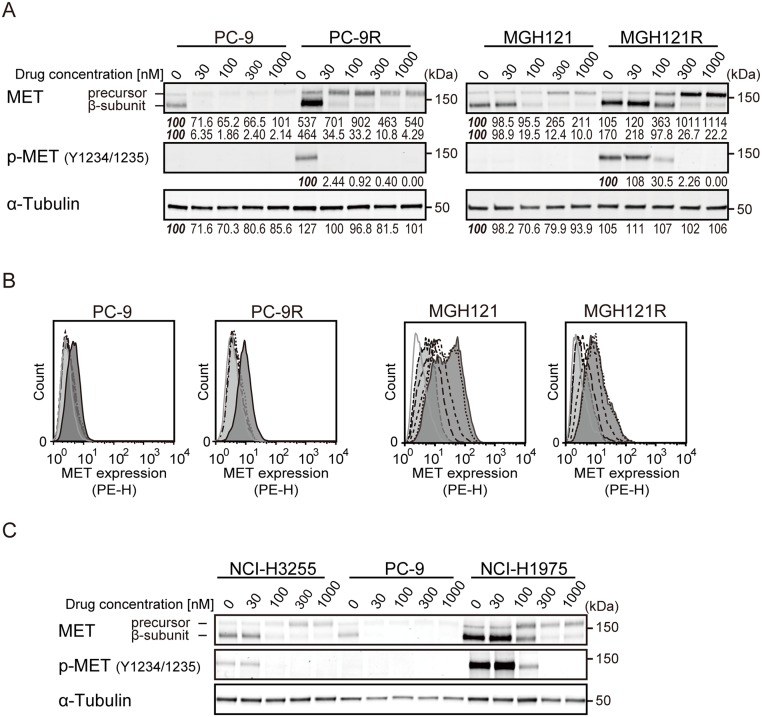
The effect of M-COPA on EGFR-bypassing MET overexpression and its processing in EGFR-TKI-resistant NSCLC cell lines (**A**) The processing and phosphorylation status of MET protein in PC-9/PC-9R cells and MGH121/MGH121R cells upon treatment with M-COPA cells at the indicated doses was examined by immunoblot analysis. Numbers indicated in each panel were relative intensities (%) of immunoreactive bands normalized to control sample (refereed as ‘*100%*’). (**B**) MET expression on the cell surface in PC-9/PC-9R cells and MGH121/MGH121R cells was measured by FCM analysis. Lines and areas are described in Figure 1A legend. (**C**) The processing and phosphorylation status of MET in EGFR-activating NSCLC cells was examined by immunoblot analysis. The processing and phosphorylation of MET protein was downregulated in MET-overexpressed EGFR-TKI-naïve NCI-H1975 cells (T790M/L858R) exhibiting a natural resistance to EGFR-TKIs.

As described above, most of the acquired resistance to EGFR-TKI was mediated by a secondary or tertiary mutation of the EGFR gene, and we have thus far demonstrated that M-COPA overcame TKI-resistance induced by such mutations. In addition, the activation of an alternative signaling pathways bypassing EGFR, i.e., the overexpression of MET, has been reported to be another mechanism of acquired resistance to EGFR-TKIs [14, 39]. MET is a receptor tyrosine kinase that functions as a receptor for hepatocyte growth factor (HGF), and the gene amplification of MET drives cells to malignant proliferation and survival [32, 40]. Because we previously demonstrated that M-COPA prevented the maturation and transport of the MET protein to the cell surface to downregulate its downstream signaling pathway in MET-amplified gastric cancer cells [33], we expected that M-COPA might also downregulate MET expression in EGFR-TKI-resistant NSCLC cells. Interestingly, immunoblot analyses revealed that MET expression was elevated in PC-9R and MGH121R cells compared with their parental PC-9 and MGH121 cells, respectively (Figure 5A). Moreover, the phosphorylated form of MET was exclusively detected in acquired resistant PC-9R and MGH121R cells. Upon treatment with M-COPA, an accumulation of the precursor form of MET and the downregulation of its mature β-subunit were observed in parallel to the downregulation of cell surface MET expression (Figure 5B). From these results, we concluded that M-COPA overcomes EGFR-TKI resistance in NSCLC cells by downregulating the cell surface expression of both mutated EGFR and MET, which might be involved in the EGFR-bypassing alternative pathway. In addition, EGFR-TKI-naïve intrinsically resistant NCI-H1975 cells also strongly expressed the mature phosphorylated form of MET, and M-COPA completely diminished MET maturation, which remained in its precursor form (Figure 5C).

### M-COPA suppresses tumor growth in NSCLC-derived tumor xenografts *in vivo*

**Figure 6:**
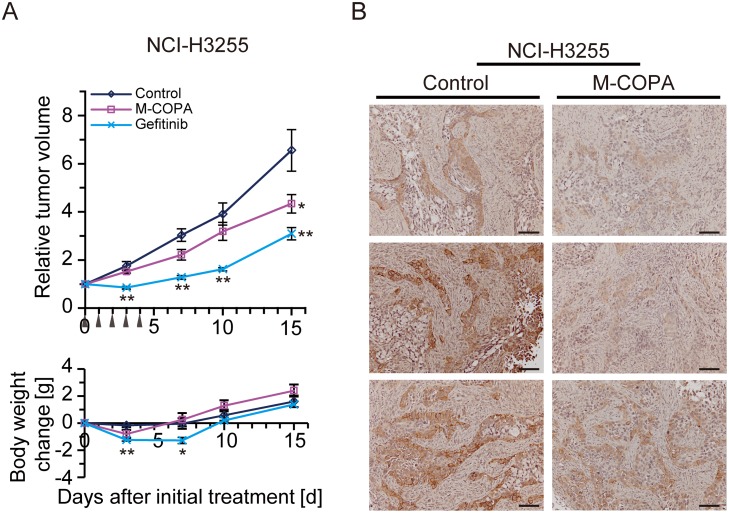
*In vivo* antitumor efficacy of M-COPA against EGFR-activated NSCLC tumors derived from subcutaneously implanted NCI-H3255 cells (**A**) Human NSCLC NCI-H3255 cells were subcutaneously inoculated into BALB/c nude mice. Tumor bearing mice were randomly divided into a control group (*n* = 6), M-COPA group (*n* = 6), and gefitinib group (*n* = 6). Mice were administered with the indicated drugs daily from day 0 to day 4, shown by arrowheads. The upper panel shows changes in the relative tumor growth ratio, while the lower panel shows body weight change. A two-sided Mann–Whitney *U*-test was used to assess the statistical significance of the antitumor efficacy in terms of relative tumor growth ratio, and Welch's *t*-test was used for body weight change on days 3, 7, 10, and 15. Asterisks represent statistically significant differences compared with the control group (^*^p < 0.05; ^**^p < 0.01) error bar, S.E. (**B**) Immunohistochemistry of tumor sections from control and M-COPA-administered mice (6 h after a single administration of M-COPA at 50 mg/kg BW). Scale bar, 100 μm.

**Figure 7:**
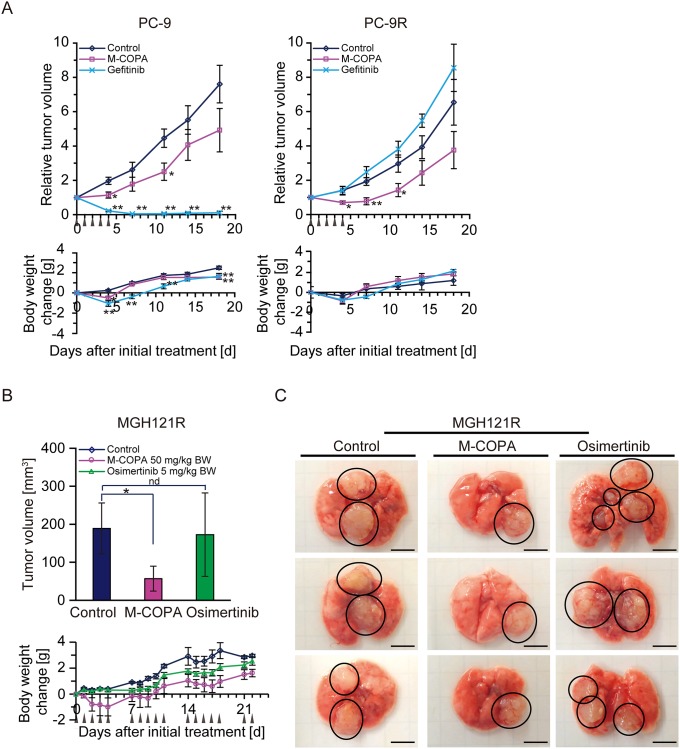
*In vivo* antitumor efficacy of M-COPA against EGFR-TKI-resistant NSCLC tumor xenografts with a secondary or/and tertiary EGFR mutation (**A**) Human NSCLC PC-9 cells and their parental gefitinib-resistant PC-9R cells were subcutaneously inoculated into BALB/c nude mice. Tumor-bearing mice were randomly divided into a control, M-COPA, and gefitinib group. Each PC-9-bearing group contained 6 mice, the PC-9R-bearing groups contained either 5 (control and M-COPA group) or 4 mice (gefitinib group). Mice were administered with the indicated drugs daily from day 0 to day 4, as shown by arrowheads. Welch's *t*-test was used to assess the statistical significance for animals inoculated with PC-9 cells, and a two-sided Mann–Whitney *U*-test was used for PC-9R cells on days 4, 7, 11, 14, and 18. Asterisks represent statistically significant differences compared with the control group (^*^p < 0.05; ^**^p < 0.01) error bar, S.E. (**B**) MGH121R cells were orthotopically injected into the left lung parenchyma. Engraftment of tumors was observed by MRI. Mice were divided into three groups: control group (*n* = 5), M-COPA group (*n* = 6), and osimertinib group (*n* = 6). At 21 days after implantation, administration was started (day 0). Mice were administered with the indicated drug using a 5 days on, 2 days off schedule, as indicated by arrowheads. On day 22, after sacrificing animals, the lung bearing tumor nodes were harvested and tumor volumes in each lung were calculated as described in the Materials and Methods. The upper panel shows the tumor size on day 22, while the lower panel shows body weight changes in nude mice. Asterisks represent statistically significant differences compared with the control group (^*^p < 0.05). nd, no difference; error bars, S.D. (**C**) Representative tumor nodes on lungs harvested from MGH121R inoculated mice at day 22. Circled areas indicate each tumor node. Scale bar, 5 mm.

Finally, we tested the antitumor efficacy of M-COPA against NSCLC xenograft models. Nude mice bearing subcutaneously xenografted NCI-H3255 tumors were administered with M-COPA (50 mg/kg BW) or gefitinib (150 mg/kg BW) daily from day 0 to day 4. Tumor growth was significantly suppressed by M-COPA without severe body weight loss; however, its antitumor activity lower compared with that of gefitinib (Figure 6A). Immunohistochemistry of tumor sections revealed that the phosphorylated form of EGFR was significantly decreased within 6 h after a single administration of M-COPA (Figure 6B). Similar results were observed in PC-9 tumors. Modest antitumor activity was observed upon the administration of M-COPA, whereas tumors exhibited a complete response to gefitinib (Figure 7A, left panel).

We next examined the antitumor effect of M-COPA against NSCLC cells harboring a TKI-resistant mutation of EGFR. First, we exploited PC-9R tumors subcutaneously xenografted into nude mice. Unlike their parental PC-9 tumors, PC-9R tumors no longer responded to gefitinib. In contrast, they exhibited a significant response to M-COPA, and this response was greater than the response against the parental PC-9 tumors (Figure 7A, right panel). Second, we examined the antitumor effect of M-COPA on MGH121R tumors. Because neither MGH121 nor MGH12R could be subcutaneously engrafted in BALB/c nude mice, we developed an orthotopic xenograft model. The reproducible and stable engraftment of MGH121R tumors, but not parental MGH121, was observed by magnetic resonance imaging at 15 days after their inoculation into the lung parenchyma (Supplementary Figure 2, data not shown). We then examined the effect of M-COPA on MGH121R orthotopic xenografts in comparison with osimertinib using a 5 days on, 2 days off schedule for 3 weeks. While the administration of osimertinib did not induce an antitumor response, M-COPA attenuated tumor progression (Supplementary Figure 2). The measurement of tumor volumes following the sacrifice of mice on day 22 clearly demonstrated smaller tumor volumes in the M-COPA-administered group compared with the control group, an effect not observed in the osimertinib-administered group (Figure 7B upper panel, Figure 7C). These results indicate that M-COPA exerts *in vivo* antitumor activity against first- and third-generation EGFR-TKI-resistant NSCLC cells xenografted into nude mice, and that this class of compound might be useful for the treatment of NSCLC patient in the clinic.

## DISCUSSION

We previously demonstrated that targeting the Golgi apparatus using an Arf1 inhibitor, M-COPA, exerted a selective antitumor effect on gastric cancers amplifying the RTK genes MET and FGFR2 by downregulating the cell surface expression of such RTKs [33]. In the present study, we demonstrated that M-COPA dispersed Golgi structure, downregulated cell surface expression of mutated EGFR and its downstream signals and exerted an antitumor effect on NSCLC cells harboring an activating EGFR mutation; however, the effect was modest when compared with EGFR-TKIs such as gefitinib. The EGFR activating mutation was reported to be a major therapeutic target in NSCLCs, and many patients who received EGFR-TKI therapy displayed an improved clinical outcome compared with those who received conventional chemotherapy [11, 30, 37]. In this context, M-COPA would not replace EGFR-TKIs as a first-line therapy in EGFR-activated NSCLC patients. The most important finding in the present study was that M-COPA exerted comparable antitumor activity in NSCLC cells with acquired TKI resistance to NSCLC cells without TKI resistance. Of note, acquired resistance was reported as a major problem for TKI therapy [9, 41, 42]. NSCLC patients who received first-generation EGFR-TKI therapy might develop resistance to EGFR-TKIs by secondary EGFR-T790M mutations. Despite the development of third-generation covalent EGFR-TKIs that inhibit the kinase activity of EGFR-T790M mutants, the generation of tertiary C797S was clinically reported and experimentally demonstrated [12, 13]. Therefore, there is an urgent need to overcome the acquired resistance to EGFR-TKIs in NSCLC patients. Recent reports, including ours, have demonstrated that overcoming the resistance to third-generation EGFR-TKIs can be accomplished experimentally using the allosteric EGFR-TKI EAI045, and the EGFR-C797S/T790M/del19 mutant-selective TKI brigatinib, which was originally developed as an ALK kinase inhibitor, in combination with the anti-EGFR monoclonal antibody cetuximab [43, 44]; however, analogous to previous findings, unknown mutations in the EGFR gene exhibiting acquired resistance to these agents can be generated. Our present study provides evidence that targeting the Golgi apparatus by M-COPA might be a promising therapeutic approach to overcome the vicious cycle of TKI resistance in EGFR-mutated NSCLC cells *via* a completely different mode of action, *i.e.*, downregulating the cell surface expression of EGFR with a TKI-resistant mutation. Moreover, our results demonstrated that M-COPA efficiently suppressed the cell surface expression and maturation of MET, which might also be involved in the acquisition of resistance to EGFT-TKIs. Indeed, the level of MET was greatly increased, and its phosphorylated form was specifically observed in both PC-9R and MGH121R, and the administration of M-COPA abolished MET activation.

This study reports the first evidence that M-COPA decreased the level of cell surface expression of EGFR harboring an activating mutation (Figure 1A), abrogated its downstream oncogenic signals represented by a reduction of the phosphorylated forms of Akt, S6, MEK and ERK (Figure 1B), and ultimately suppressed tumor growth in EGFR-activated NSCLC cell lines (Figure 2A). From these results, we concluded that the inhibition of EGFR-cell surface expression as a consequence of Golgi dispersion is the main mechanism by which M-COPA exerts its antitumor effects on NSCLC cells. However, NCI-H226, which has wild type EGFR, displayed a favorable sensitivity to M-COPA (Figure 2A). The reason why M-COPA was effective against NCI-H226 cells remains unclear. Although we demonstrated that neither ErbB2, ErbB3 nor ErbB4 were overexpressed in this cell line (data not shown), the involvement of other RTKs or other cell surface proteins should be considered. BFA, another Golgi disruptor, is a potent inducer of endoplasmic reticulum (ER) stress and unfolded protein response (UPR) [45], and similar activity to M-COPA is expected. Therefore, the involvement of UPR in the antitumor effect of M-COPA should also be considered.

In this study, we demonstrated that M-COPA exerts *in vivo* antitumor activity against first- and third-generation EGFR-TKI-resistant NSCLC cells subcutaneously- or orthotopically xenografted into nude mice (Figure 7). These results clearly represented the preclinical proof of concept for the use of M-COPA to overcome EGFR-TKI resistance. However, antitumor activity of M-COPA was lower than that of gefitinib in EGFR-TKI naïve PC-9 and NCI-H3255 xenograft models, as mentioned earlier. It will be of great interest to investigate the combinatorial effect of M-COPA and existing chemotherapeutic agents such as paclitaxel and cisplatin on these EGFR-mutated NSCLC cells.

In agreement with our previous report [33], the daily administration of M-COPA for 5 consecutive days resulted in slight weight loss in recipient mice, and weight loss was alleviated after stopping administration. Furthermore, M-COPA administration using a 5 days on, 2 days off schedule for 3 weeks did not result in extensive body weight loss (Figure 7B and 7C). The results indicated that this compound was well tolerated and could be administered for longer periods to maintain its antitumor effect. Considering the global presence of Golgi apparatus in both normal and tumor cells, it was interesting that M-COPA selectively suppressed tumor growth without serious adverse effects. We postulated that the effect of M-COPA on cancer and normal cells could be determined by two factors: (1) their survival and growth is dependent on a cell surface protein like RTK, and (2) transport to cell surface and/or posttranslational modification of above-mentioned protein is dependent on Golgi apparatus and can be blocked by M-COPA. Indeed, tumor cells addicted to aberrant RTK expression such as MET-amplified GCs and EGFR-mutated NSCLCs were highly sensitive to M-COPA, and these RTKs were effectively downregulated by M-COPA treatment. In contrast, animal experiments indicated that M-COPA was well tolerated and did not cause severe body weight loss, suggesting its low toxicity in normal cells. Therefore, survival of normal cells seemed to be independent of cell surface expression of M-COPA-responsive protein. Of note, M-COPA's effect on cell surface expression seemed to vary depending on proteins. For example, P-glycoprotein was not at all downregulated upon M-COPA treatment, while BCRP was downregulated effectively [33]. More interestingly, wild-type EGFR was not much affected by M-COPA, while mutated EGFR was greatly downregulated. These differences could account for M-COPA's selective cytotoxicity. We have not yet determined the mechanism by which these differences caused, but we supposed that the differences in coated vesicles (e.g., COPI-coated or clathrin-coated vesicles) may cause different response to M-COPA. Further studies are needed to clarify the mechanism by which M-COPA exerts selective toxicity.

In conclusion, we demonstrated that M-COPA disrupted Golgi structure, inhibited the transport of EGFR protein harboring an activating mutation to the cell surface, attenuated aberrant EGFR signaling and exerted a preferential antitumor activity against EGFR-activated NSCLC cells. These events were even more prominent in cells with acquired resistance to first- and third-generation EGFR-TKIs compared with those without acquired resistance. In these cells, the overexpression and phosphorylated MET was detected, but was efficiently abolished by M-COPA treatment. Animal experiments confirmed the *in vivo* antitumor effect of M-COPA on TKI-resistant tumors. The present results provide preclinical evidence that a Golgi-targeted drug might be a novel therapeutic modality with a unique mode of action to overcome TKI resistance in NSCLC patients.

## MATERIALS AND METHODS

### Chemicals

M-COPA (formerly termed ‘*AMF-26*’, chemical name: (2*E*,4*E*)-5-((1*S*,2*S*,4a*R*,6*R*,7*S*,8*S*,8a*S*)-7-hydroxy-2,6,8-trimethyl-1,2,4a,5,6,7,8,8a-octahydronaphthalen-1-yl)-2-methyl-*N*-(pyridin-3-ylmethyl)penta-2,4-dienamide) was totally synthesized by Eisai Inc. (Andover, MA) according to our previously established methods [46]. Gemcitabine, paclitaxel, and cisplatin were purchased from Eli Lilly and Company (Indianapolis, IN), Sigma-Aldrich Co. LLC. (St. Louis, MO), and Nippon Kayaku Co., Ltd., (Tokyo, Japan), respectively. Gefitinib, erlotinib, and osimertinib for *in vitro* studies were purchased from Selleck Chemicals (Houston, TX). For animal experiments, gefitinib tablets (Iressa^®^ Tablets 250 mg) were purchased from AstraZeneca Japan (Osaka, Japan). Osimertinib mesylate was purchased from ChemScene (Monmouth Junction, NJ).

### Cell lines and cell culture

Five NSCLC cell lines (NCI-H23, NCI-H522, NCI-H226, NCI-H460, and A549) are components of the JFCR39 panel of human cancer cell lines described previously [47, 48]. NCI-H23, NCI-H522, NCI-H226, NCI-H460 were dispensed from the National Cancer Institute (NCI), and A549 was purchased from the American Type Culture Collection (Manassas, VA). NCI-H3255 (EGFR-L858R) and NCI-H1975 (EGFR-T790M/L858R) were purchased from the American Type Culture Collection (ATCC). PC-9 (EGFR-del19) was a kind gift from Dr. Kazuto Nishio (Kinki University Faculty of Medicine, Osaka, Japan) [35]. MGH121 (EGFR-T790M/del19) and MGH121-resistant-2 (EGFR-C797S/T790M/del19; or ‘*MGH121R*’, hereafter) were described previously [13, 44] and were a kind gift from Dr. Jeffrey A. Engelman (Massachusetts General Hospital, MA). Cell lines were cultured in RPMI 1640 medium (Wako Pure Chemical Industries, Osaka, Japan) supplemented with 5% (*v/v*) fetal bovine serum (Cell Culture Bioscience, Tokyo, Japan), 1% (v/v) antibiotic-antimycotic solution (100×; Sigma-Aldrich), and 100 μg/ml kanamycin (Meiji Seika Pharma Co., Ltd., Tokyo Japan) in a humidified atmosphere including 5% CO_2_ at 37°C. Authentication of cell lines except MGH121 and MGH121R was performed by short tandem repeat (STR) analysis using PowerPlex16 Systems (Promega, Madison, WI) at BEX Co., Ltd., (Tokyo, Japan) according to the manufacturer's instructions. Finally, the STR profiles were checked against the reference databases of the European Collection of Authenticated Cell Cultures (ECACC), ATCC, and Japanese Collection of Research Bioresources (JCRB).

### Generating a gefitinib-resistant cell line PC-9R *in vitro*

To generate resistance to gefitinib, PC-9 parental cells were grown in increasing doses of gefitinib starting at 1 nM and increasing incrementally to 1000 nM once the cells began to grow through the given dose.

### DNA sequencing and allele quantification assay

Genomic DNA (gDNA) was prepared from PC-9 parental and resistant (PC-9R) cells using the NucleoBond CB kit (Macherey-Nagel GmBH & Co., Düren, Germany) according to the manufacture's protocol. Preparation of sequence libraries and analysis of the next generation DNA sequencing (NGS) were conducted by Takara Bio Inc., (Shiga, Japan). Briefly, the genomic library was prepared from randomly cleaved gDNA fragments by use of an Acoustic Solubilizer (Covaris, Inc., Woburn, MA). Then, the fragments were PCR amplified to create sequencing libraries by using SureSelect XT reagent Kit and SureSelect Human ALL Exon Kit V5 (Agilent Technologies, Santa Clara, CA). NGS was performed by use of HiSeq 2500 (Illumina Inc., San Diego, CA). Data was analyzed by Genedata Expressionist (Genedata AG, Basel, Switzerland). The Integrative Genomics Viewer (IGV, Broad Institute, Cambridge, MA) was used for visualization of the genomic alignment data.

### Analysis of cell growth inhibition

The inhibition of cell growth was assessed by measuring changes in total cellular protein in a culture of each NSCLC cell line after 48 h of drug treatment by use of a sulforhodamine B (SRB) assay [49]. Positive values represent the net protein increase before and after drug exposure (% of control growth) and negative values represent cell death (protein amount after 48 h-exposure (%) of control cells at the start of drug exposure). The drug concentration required for 50% reduction in net protein increase (GI_50_) was calculated as described previously [47, 49, 50].

### Flow cytometric (FCM) analysis

Cells were incubated with M-COPA at the indicated concentrations for 24 h, washed with ice-cold PBS and stained with antibodies against human EGFR or MET conjugated with phycoerythrin (PE). Detailed information about the antibodies used in this study is shown in the Supplementary Table 1. Cells were washed three times with ice-cold PBS and stained with propidium iodide (1 μg/mL; Sigma-Aldrich). The fluorescence intensity of cell surface EGFR was measured by FCM (FACS Calibur or FACS Verse, Becton, Dickinson and Company, Franklin Lakes, NJ). The data was analyzed by FlowJo software (FlowJo LLC., Ashland, OR).

### Western blot analysis

Cells were incubated with M-COPA for 24 h, and then lysed as described previously [51]. Proteins in cell lysates were separated by 4–15% sodium dodecyl sulfate–polyacrylamide gel (Bio-Rad Laboratories, Hercules, CA) electrophoresis, followed by electroblotting onto a nitrocellulose membrane (Bio-Rad Laboratories). Primary and secondary antibodies used for immunostaining are listed in the Supplementary Table 1. Immunoreactive bands were visualized and quantitated by using the ODYSSEY^®^ CLx Infrared Imaging System (LI-COR Biosciences, Lincoln, NE).

### Animal experiments

Animal care and treatment was performed in accordance with the guidelines of the Animal Use and Care Committee of the Japanese Foundation for Cancer Research, and conformed to the NIH Guide for the Care and Use of Laboratory Animals. Female nude mice of BALB/c genetic background were purchased from Charles River Laboratories Japan Inc., (Yokohama, Japan), maintained under specific pathogen-free conditions and provided with sterile food and water *ad libitum*. The antitumor effect of M-COPA was tested *in vivo* against mice bearing xenografts derived from subcutaneously implanted human NSCLC cell lines, NCI-H3255, PC-9 and PC-9R. For orthotopic implantation models, MGH121R cells (2.3 × 10^6^ cells/mouse) were resuspended in Matrigel^®^ Basement Membrane Matrix (Corning Incorporated, Corning, NY) diluted in Gibco™ Hank's Balanced Salt Solution (Life Technologies, Carlsbad, CA) (1:2) in a volume of 50 μL, and the cell suspension was injected into the left lung parenchyma. After maintenance for two weeks, the engraftment of tumors was confirmed by a MR VivoLVA^®^ 1508 magnetic resonance imaging system (1.5T, DS Pharma Biomedical, Osaka, Japan). To obtain a magnetic resonance imaging (MRI) map, we used a protocol including the 3D T1-weighted Gradient Echo sequence (repetition time (TR) = 50.0 msec, echo time (TE) = 6.0 msec). The experimental group of mice was orally administered a dose of M-COPA (50 mg/kg of body weight [BW]), gefitinib (150 mg/kg of BW in NCI-H3255 or PC-9/PC-9R bearing mice) or osimertinib (5 mg/kg of BW in MGH121R bearing mice) daily according to the schedule indicated for each experiment. The control group of mice was orally administered 0.15 N hydrochloride acid solution. The tumor volume of tumor-bearing mice was measured as described previously [51]. Briefly, the length (L) and width (W) of the tumor mass were measured by calipers (subcutaneous model) or by digital image processing using ImageJ software (National Institute of Health, Bethesda, MD, for the orthotopic model), and the tumor volume (TV) was calculated as follows: TV = (L × W^2^)/2. To assess toxicity, the body weights of the tumor-bearing mice were measured.

### Immunohistochemistry

Formalin fixed, paraffin embedded tissue sections (4-μm thick) were deparaffinized in xylene and passed through a series of graded alcohols to water. Then, antigens were retrieved in Dako Real Target Retrieval Solution (pH 6.0) at 95°C for 40 min. Sections were blocked with 3% H_2_O_2_ and 1% goat serum before incubation with a primary antibody at 4°C overnight. By using the Dako EnVision™ Detection System/HRP, Rabbit/Mouse (DAB+) (Agilent Technologies Company, Santa Clara, CA), sections were incubated with horseradish peroxidase (HRP)-conjugated polymer secondary antibodies, and thereafter peroxidase activity was visualized by diaminobenzidine reaction according to the manufacturer's instructions. The sections were counterstained with hematoxylin (Dako). The immuno-stained specimens were imaged using a microscope (BX41; Olympus Corp., Tokyo, Japan) with 20× NA 0.50 objective, and DP2-BSW Software (Olympus Corp.).

### Immunofluorescent staining of Golgi apparatus

Immunofluorescent staining of Golgi apparatus was performed as described previously [21]. Briefly, cells were treated with M-COPA for 1 h, and fixed and permeabilized. Golgi apparatus were visualized by using primary antibody against Golgi Brefeldin A Resistant Guanine Nucleotide Exchange Factor 1, GBF1 (BD Transduction Lab., Franklin Lakes, NJ) and fluorescent secondary antibody. The immunostained cells were imaged using a fluorescent microscope IX81 (Olympus Corp., Tokyo, Japan) with a ×60, NA 0.90 objective or with a ×20, NA 0.75 objective, and MetaMorph Software (Molecular Devices, Sunnyvale, CA).

### Statistical analysis

The statistical significance of the antitumor efficacy of M-COPA in terms of relative tumor growth ratios was assessed by a Mann–Whitney *U*-test or Welch's *t*-test as indicated in respective experiments. The statistical significance of the difference in tumor volume between the control group and drug-treated group in the orthotopically xenografted model was assessed by Welch's *t*-test. All statistical tests were two-sided.

## SUPPLEMENTARY MATERIALS FIGURES AND TABLE



## Abbreviations

arf1: ADP ribosylation factor 1
BFA: brefeldin A
COP: coatomer protein complex
EC_50_: 50% effective concentration value
EGFR: epidermal growth factor receptor
ER: endoplasmic reticulum
FGFR: fibroblast growth factor receptor
GI_50_: 50% growth inhibitory concentration value
HGF: hepatocyte growth factor
M-COPA: methylcoprophilinamide
MET: hepatocyte growth factor receptor
NSCLC: non-small cell lung cancer
RTK: receptor tyrosine kinase
TKI: tyrosine kinase inhibitor.
