# Supplementary material for: Targeting the Golgi apparatus to overcome acquired resistance of non-small cell lung cancer cells to EGFR tyrosine kinase inhibitors
Source: Oncotarget. 2017 Dec 6;9(2):1641–55. doi: 10.18632/oncotarget.22895 (PMC5788588; doi:10.18632/oncotarget.22895)
Supplement: Supplementary file 1 [file oncotarget-09-1641-s001.pdf]

Targeting the Golgi apparatus to overcome acquired resistance of non-small cell lung cancer cells to EGFR tyrosine kinase inhibitors

SUPPLEMENTARY MATERIALS

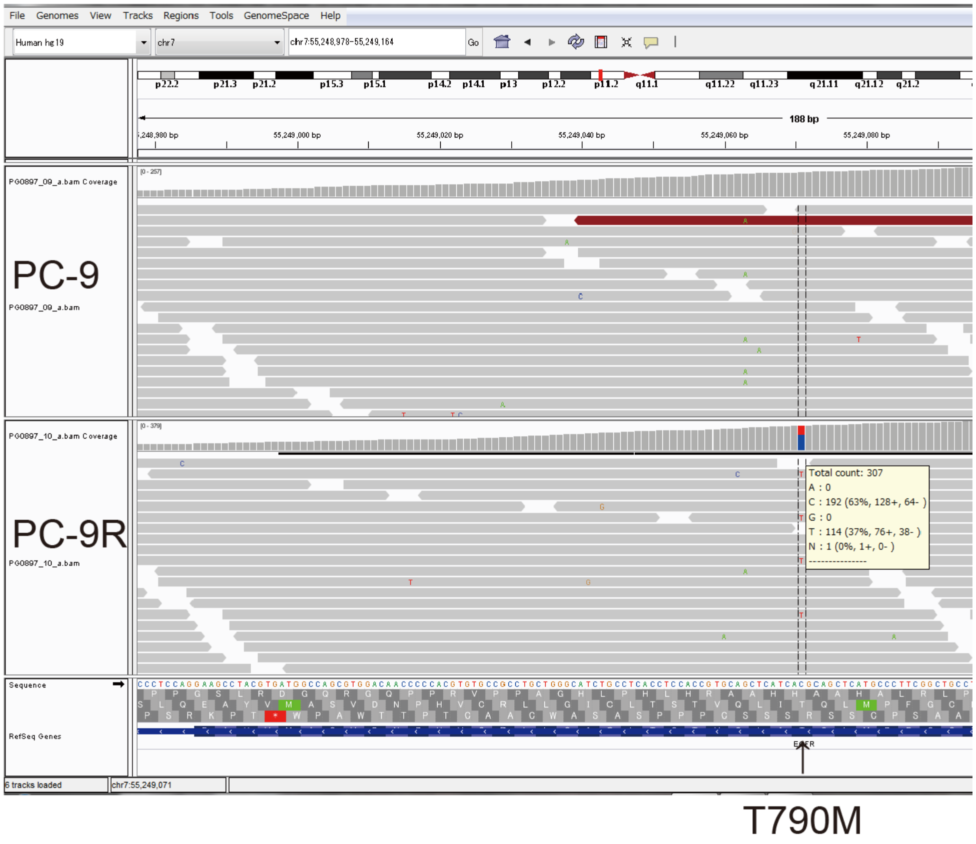

Supplementary Figure 1: Detection of EGFR T790M mutation in gefitinib-resistant PC-9R cells. Targeted NGS identified the T790M ('ACG' to 'ATG') mutation in the gDNA from PC-9R cells. The allele frequency of 'ATG' is 114/307 (37%) in PC-9R, but no such mutation was detected in the parental PC-9 cells.

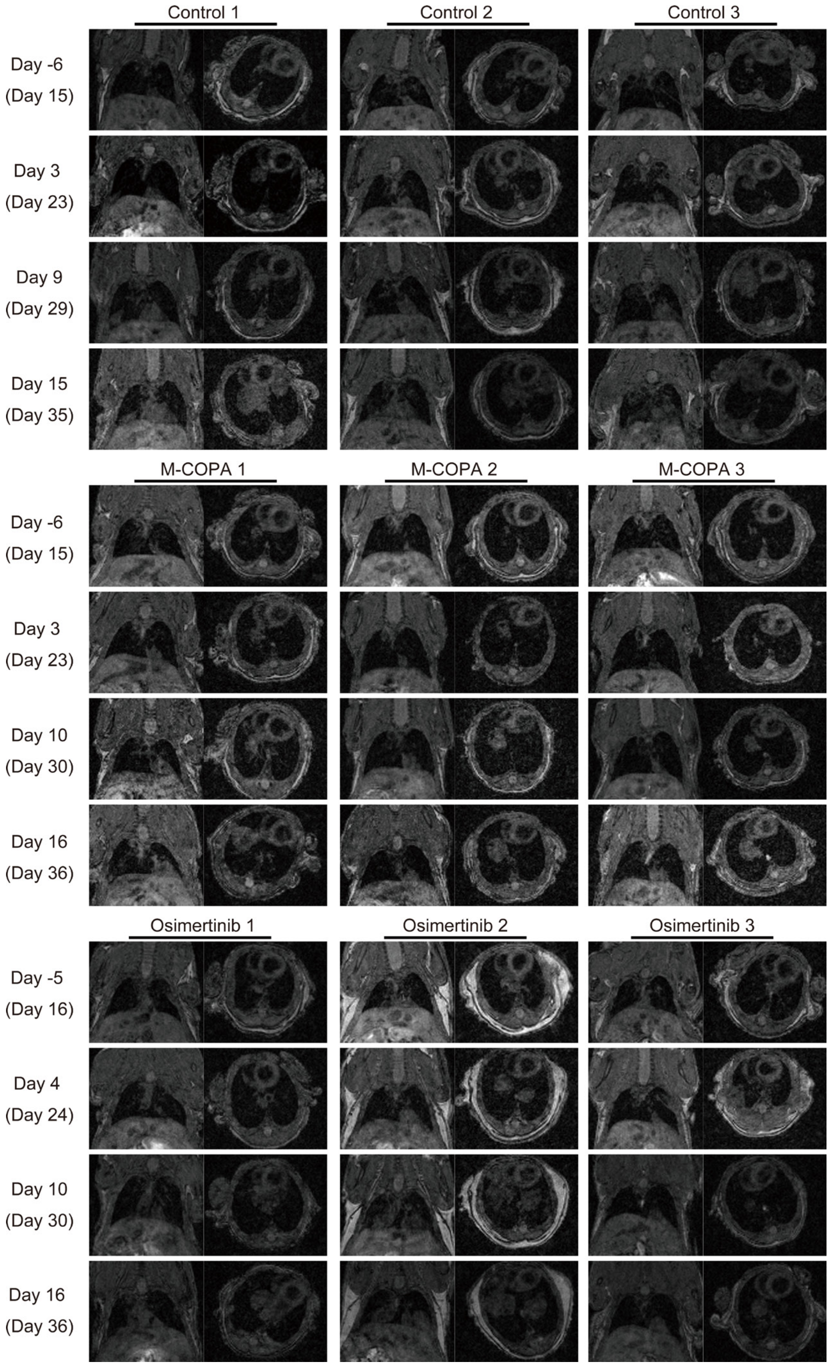

Supplementary Figure 2: *In vivo* imaging of MGH121R tumor xenografts orthotopically implanted in nude mice and antitumor efficacy of M-COPA compared with osimertinib. Engraftment of tumors implanted in the left lung parenchyma and tumor progression was monitored by MRI, using a protocol including a 3D T1-weighted Gradient Echo sequence. MRI images were obtained on the indicated days from the initiation of drug administration (day 0) indicated on the left of each image. Elapsed days from the day of tumor inoculation are indicated in parentheses.

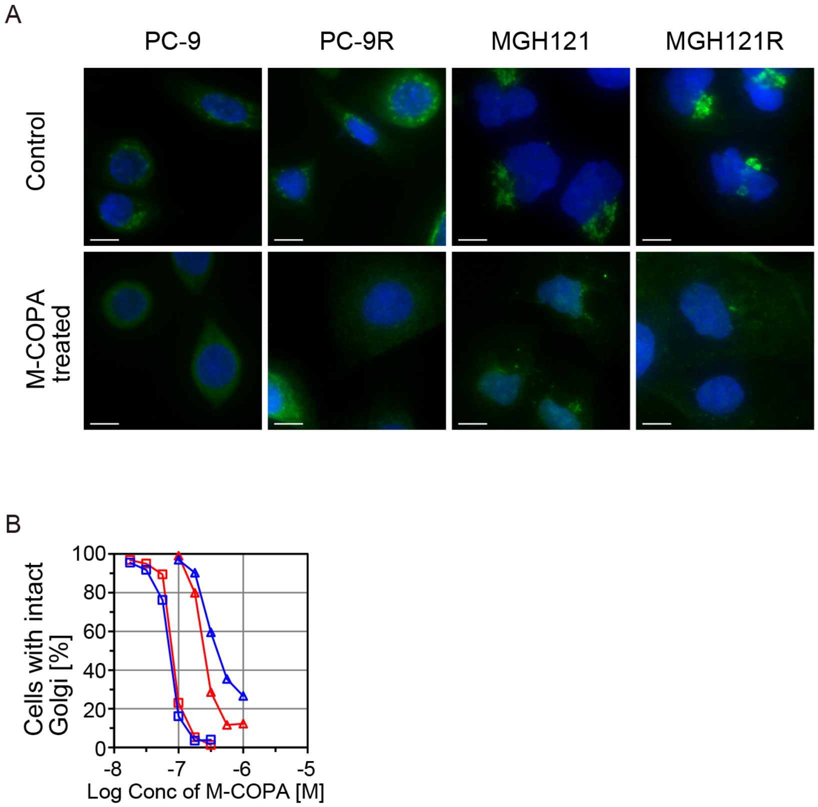

Supplementary Figure 3: Dispersion of Golgi apparatus upon M-COPA treatment in EGFR-mutated human NSCLC cell lines. The effect of M-COPA on Golgi structure in NSCLC cells was examined by immunofluorescence imaging. (**A**) Representative immunofluorescence images of NSCLC cells. Cells were treated with M-COPA at a concentration of 100 nM (PC-9 and PC-9R) or 300 nM (MGH121 and MGH121R) for 1h. Golgi apparatus and nuclei were visualized by using a monoclonal antibody against a cis-Golgi marker, GBF1, fluorescent secondary antibody and DAPI. M-COPA dispersed Golgi apparatus in all of NSCLC cell lines examined. Scale bars: 10 nm. (**B**) Dosedependent effect of M-COPA on Golgi integrity in EGFR-TKI-resistant PC-9R and MGH121R cells and their parental cells. Cells were treated with M-COPA at the indicated concentrations and Golgi apparatus and nuclei were visualized. The percentage of cells with intact Golgi at each dose were calculated, and doseresponse curves were drawn for the respective cell lines. Using the doseresponse. curves, ECR_{50} values were calculated. Symbols indicate the following: red square, PC-9; blue square, PC-9R; red triangle, MGH121; blue triangle, MGH121R.

Supplementary Table 1: List of the primary and secondary antibodies used in this study

Antibody	Clone	Cross reactivity	Conjugate	Application	Company	Catalog No.
EGFR rabbit mAb	D38B1	H M Mk	Unconjugated	WB	CST	#4267
p-EGFR (Tyr1068) rabbit mAb	D7A5	H M R Mk	Unconjugated	WB, IHC	CST	#3777
Akt (pan) rabbit mAb	C67E7	H M R Mk Dm	Unconjugated	WB	CST	#4691
p-Akt (Ser473) rabbit mAb	D9E	H M R Hm Mk Dm Z B	Unconjugated	WB	CST	#4060
p-Akt (Thr308) rabbit mAb	C31E5E	H M R Hm, Mk	Unconjugated	WB	CST	#2965
S6 Ribosomal Protein rabbit mAb	5G10	H M R Mk	Unconjugated	WB	CST	#2217
p-S6 (Ser235/236) rabbit mAb	D57.2.2E	H M R Mk Mi Sc	Unconjugated	WB	CST	#4858
p-S6 (Ser240/244) rabbit mAb	D68F8	H M R Mk	Unconjugated	WB	CST	#5364
MEK1/2 rabbit mAb	D1A5	H M R Mk Dm	Unconjugated	WB	CST	#8727
p-MEK1/2 (Ser217/221) rabbot, Ab		H M R Mk, Sc	Unconjugated	WB	CST	#9121
ERK1/2 rabbit mAb	137F5	H M R Hm Mk Mi Dm Z B Dg Pg Ce (C)	Unconjugated	WB	CST	#4695
p-ERK1/2 (Thr202/204) rabbit mAb	D13.14.4E	H M R Hm Mk Mi Dm Z B Dg Pg Sc	Unconjugated	WB	CST	#4695
Met rabbit mAb	D1C2	H	Unconjugated	WB	CST	#8198
p-Met (Tyr1234/1235) rabbit mAb	D26	H M R	Unconjugated	WB	CST	#3077
a-tubulin mouse mAb	B-5-1-2	H M R Mk B	Unconjugated	WB	Sigma	T5168
IRDye 800CW goat anti-M IgG (H+L)		M	IRDye® 800CW	WB	LI-COR	926-32210
AlexaFluor 680 goat anti-R IgG (H+L)		R	AlexaFluor 680	WB	Molecular Probe	A-21109
EGFR mAb, mouse IgG2b	EGFR.1	H	Phycoerythrin	FCM	BD	555997
IgG2b, k isotype control	27-35	M	Phycoerythrin	FCM	BD	555743
HGFR/Met mAb, mouse IgG1	95106	H	Phycoerythrin	FCM	R&D	FAB3582P
IgG1, k isotype control	MOPC-21	M	Phycoerythrin	FCM	BD	555749
GBF1 mouse mAb	Clone 25	M	Unconjugated	FC	BD	612116
AlexaFluor 488 goat anti-M IgG (H+L)		M	AlexaFluor 488	FC	Molecular Probe	A-11029
